# Production-related contaminants (pesticides, antibiotics and hormones) in organic and conventionally produced milk samples sold in the USA

**DOI:** 10.1017/S136898001900106X

**Published:** 2019-06-26

**Authors:** Jean A Welsh, Hayley Braun, Nicole Brown, Caroline Um, Karen Ehret, Janet Figueroa, Dana Boyd Barr

**Affiliations:** 1 Department of Pediatrics, Emory University School of Medicine, W-450 Health Sciences Research Building – Room E400, 1760 Haygood Drive NE, Atlanta, GA 30322, USA; 2 Wellness Department, Children’s Healthcare of Atlanta, Atlanta, GA, USA; 3 Department of Pharmacology, Emory University School of Medicine, Atlanta, GA, USA; 4 Department of Epidemiology, Rollins School of Public Health, Atlanta, GA, USA; 5 Department of Environmental Health, Rollins School of Public Health, Atlanta, GA, USA

**Keywords:** Milk, Organic, Production, Contaminants, Pesticides, Antibiotics, Hormones, Insulin-like growth factor 1

## Abstract

**Objective:**

Consumption of cow’s milk, which is associated with diet and health benefits, has decreased in the USA. The simultaneous increase in demand for more costly organic milk suggests consumer concern about exposure to production-related contaminants may be contributing to this decline. We sought to determine if contaminant levels differ by the production method used.

**Design:**

Half-gallon containers of organic and conventional milk (four each) were collected by volunteers in each of nine US regions and shipped on ice for analysis. Pesticide, antibiotic and hormone (bovine growth hormone (bGH), bGH-associated insulin-like growth factor 1 (IGF-1)) residues were measured using liquid or gas chromatography coupled to mass or tandem mass spectrometry. Levels were compared against established federal limits and by production method.

**Setting:**

Laboratory analysis of retail milk samples.

**Results:**

Current-use pesticides (5/15 tested) and antibiotics (5/13 tested) were detected in several conventional (26–60 %; *n* 35) but not in organic (*n* 34) samples. Among the conventional samples, residue levels exceeded federal limits for amoxicillin in one sample (3 %) and in multiple samples for sulfamethazine (37 %) and sulfathiazole (26 %). Median bGH and IGF-1 concentrations in conventional milk were 9·8 and 3·5 ng/ml, respectively, twenty and three times that in organic samples (*P* < 0·0001).

**Conclusions:**

Current-use antibiotics and pesticides were undetectable in organic but prevalent in conventionally produced milk samples, with multiple samples exceeding federal limits. Higher bGH and IGF-1 levels in conventional milk suggest the presence of synthetic growth hormone. Further research is needed to understand the impact of these differences, if any, on consumers.

Milk consumption is associated with better diet quality and improved health^(^^1^^–^^5^^)^. Despite expert dietary guidance encouraging its consumption^(^^6^^)^, milk intake has been decreasing in the USA^(^^7^^)^. At the same time, the demand for milk produced organically has increased^(^^8^^)^. These trends suggest that concern about exposure to production-related chemicals including pesticides, antibiotics and growth hormones is playing a role in the decline of conventional milk intake^(^^8^^,^^9^^)^.

Pesticides are widely used in US food production to control pests, weeds, etc. in crops and to protect cattle from insects^(^^10^^)^. Research suggests that with sufficient exposure, some pesticides may lower birth weight, contribute to delayed motor and neurological development^(^^11^^)^, and increase cancer risk^(^^12^^)^. The maximum residue limit or tolerance limit in food is the amount of pesticide residue allowed to remain in or on a food^(^^13^^)^. These limits, based on the toxicity, frequency, amount of application and potential routes of exposure, have been developed by the US Environmental Protection Agency^(^^14^^)^. While these limits take into consideration the dietary differences and needs between adults and children, information about pesticide exposure and its related risks is very limited. Little is known about the real-life, often prolonged exposure to combinations of pesticides that may compound any effect^(^^15^^,^^16^^)^. Also unknown is the extent to which consumers are exposed to pesticides in milk and whether exposure differs when the milk consumed is produced using organic *v*. conventional methods. Luzardo *et al.* tested samples of milk sold in Spain and found low levels of the persistent pesticides organochlorines and polychlorobiphenyls in both organic and conventionally produced samples^(^^17^^)^, but no similar studies have been done to assess exposure to pesticides, either persistent or current-use pesticides, in milk produced in the USA.

Antibiotics are also commonly used in food production^(^^9^^)^. They are used prophylactically as well as to treat infections. They are also used to promote growth in animals raised for food, though their use for this purpose is expected to be more limited since new FDA rules were adopted in 2013^(^^9^^,^^18^^)^. The presence of antibiotics in the food supply has raised concerns about their possible role in increasing antibiotic resistance and hypersensitivity reactions^(^^9^^,^^19^^,^^20^^)^. Tolerance levels for antibiotics used in food production have been established by the US Food and Drug Administration (FDA)^(^^21^^,^^22^^)^. A drug, or its metabolites, is considered safe at levels believed to have little risk of toxicity or if it has been determined to be biologically inactive^(^^22^^)^. To help limit exposure to drug residues in milk, every tanker-truck entering a dairy processing plant is tested, although that testing is done only for only a small proportion of the antibiotics being used^(^^22^^–^^24^^)^. Only one known study, done a decade ago, has compared antibiotic residues in organic *v*. conventionally produced milk^(^^24^^)^.

Bovine growth hormone (bGH), also known as bovine somatotropin, is a protein produced normally by the pituitary gland of cows and other mammals that regulates the production of milk^(^^25^^)^. In 1993, the FDA approved the use of synthetic or recombinant bovine growth hormone (rbGH) by the dairy industry but concerns regarding its safety persist^(^^26^^)^. These concerns include the possible impact of rbGH’s stimulation of insulin-like growth factor 1 (IGF-1) production in man and evidence suggesting that dairy cows treated with rbGH have more frequent infections^(^^26^^,^^27^^)^. More treatment of infections increases the exposure to antibiotics, and raises the risk of antibiotic resistance, among milk consumers^(^^28^^)^. While the evidence is mixed, studies have associated higher IGF-1 levels in humans with increased risk of cancer^(^^29^^–^^32^^)^ and have raised concerns about a possible influence on immune response^(^^33^^)^ and in the growth and development in children^(^^34^^–^^36^^)^. Previous studies have concluded that, because growth hormone is species-specific and it is degraded in digestion, it is unlikely to have a biological impact on milk consumers^(^^37^^)^. More recent evidence of increased growth hormone-related antibodies in the circulation of rodents given bGH orally has cast some doubt on that conclusion^(^^28^^,^^35^^)^.

Given the prominent role of milk in the US diet, particularly in the diets of children^(^^38^^)^ and those whose consumption continues into adulthood^(^^39^^)^, the present study was done to assess the extent to which pesticides, antibiotics and synthetic hormones are present in retail milk and to determine how levels compare when produced using organic *v*. conventional methods.

## Methods

### Sample collection

**Fig. 1:**
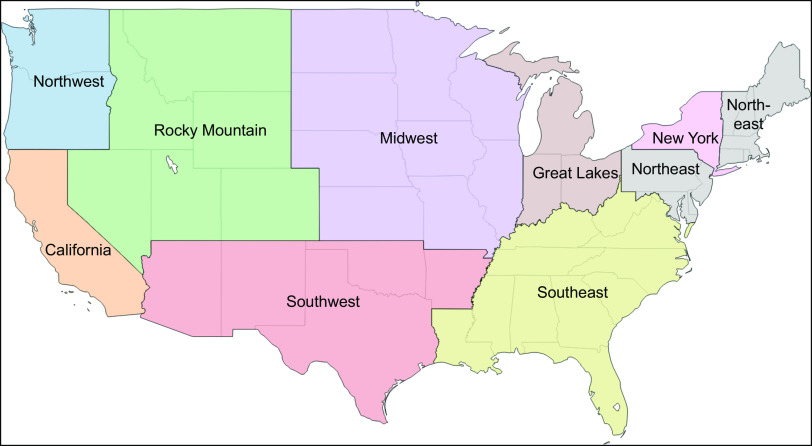
Map indicating the boundaries of each of the nine continental US regions from which a set of milk samples (three different brands of organically produced 2 % milk, three different brands of conventionally produced 2 % milk, and one each of organic and conventional whole milk) were collected in August 2015

Milk samples were collected in August 2015 from each of nine regions dividing the continental USA. Regional boundaries were those specified on a map made publicly available by one milk distributor with sales nationally (see Fig. 1)^(^^40^^)^. The selection of the specific collection site within each region was based on the location of the first volunteer shopper identified by the research team (with the assistance of friends, family and co-workers) who agreed to procure the samples and ensure that they were properly shipped to the laboratory. In each region (Table 1), eight half-gallon (1·89 litre) milk cartons were obtained from one or more retail stores selected by the volunteer for their convenience. This included six cartons of 2 % milk, the type most commonly consumed by US children^(^^7^^)^, three of which were labelled as different US Department of Agriculture-certified organic brands and three labelled as different conventional brands. In addition, given the known lipophilic nature of some pesticides, two samples of whole milk, one US Department of Agriculture-certified organic and one conventional, were obtained. Flavored and other specialty milks were excluded. All samples were shipped overnight in their original sealed containers in a single cooler and delivered on ice to Rollins School of Public Health at Emory University in Atlanta, GA. Samples were processed and labelled using a study-generated identification number to ensure blinding during laboratory analysis and stored at −20°C until analysis.

**Table 1:** Origin of milk samples available for analysis

		Number of 2 % milk samples		Number of whole milk samples		
Region	City	Organic	Conventional	Organic	Conventional	Total
California	Oceanside, CA	3	3	1	1	8
Great Lakes	Grand Rapids, MI	3	3	1	1	8
Midwest	Davenport, IA	3	3	1	1	8
New England	West Orange, NJ	3	3	1	1	8
New York	Brooklyn, NY	3	2	1	1	7
Northwest	The Dalles, OR	3	3	1	1	8
Rocky Mountain	St. George, UT	1	3	1	1	6
Southeast	Atlanta, GA	3	3	1	1	8
Southwest	Buckeye, AZ	3	3	1	1	8
Total		25	26	9	9	69

### Laboratory analysis

Milk samples were spiked with isotopically labelled internal standards, when available, subjected to liquid–liquid or solid-phase extraction, and pre-concentrated. Samples were tested for the residues of a number of commonly used pesticides and antibiotics, as determined in consultation with dairy industry experts, using previously validated methods^(^^41^^)^. Pesticide analysis was conducted via GC–MS/MS with stable isotope (^13^C) dilution quantification. Isotopically labelled internal standards were obtained from Cambridge Isotope Laboratories (Tewksbury, MA, USA) and unlabelled standards were obtained from Sigma Aldrich (St. Louis, MO, USA). The limit of detection (LOD) for each analyte varied but was in the low pg/ml, ranging from 0·02 pg/ml for atrazine to 20 pg/ml for chlorpyrifos and cypermethrin (Table 3). Analysis for antibiotic residues was performed via HPLC–high-resolution linear ion trap (LTQ Orbitrap; Thermo Scientific, San Jose, CA, USA) where the LTQ accumulates, isolates and fragments ions for MS/MS confirmation. Isotopically labelled internal standards were not available so external calibration was performed for quantification. Standards were purchased from Sigma-Aldrich. The LOD for antibiotics was 1 ng/ml (Table 3). Hormones were measured using a slight modification of the method of Kay *et al.*^(^^42^^)^ to allow for milk analysis using ultraperformance liquid chromatography–high-resolution MS using a Velos LTQ Orbitrap (Thermo Scientific). The LOD for bGH and IGF-1 were 0·40 and 0·10 ng/ml, respectively. Similar to antibiotics, external calibration without stable isotopes was used for quantification. All studies included a concurrent analysis of positive and negative controls (10 %) to ensure method validity. Positive controls consisted of pooled organic cow’s milk that was spiked with the target compounds at a concentration that was 10× the method LOD. These milk samples also contained residual levels of the target chemicals and so could not be used alone for method blanks. Negative controls included a simulated milk matrix consisting of water with 6 % (v/v) of vegetable oil and a pooled milk sample with the lowest possible analyte concentrations. Soya-based formulas were tested for blank materials but they did not appropriately mimic the recovery of the analytes from the milk as did the oil:water solution. In addition, all hormone analyses were performed in duplicate to ensure data accuracy.

### Statistical analysis

The proportions of milk samples with detectable levels of each of the pesticides and antibiotics were calculated and Fisher’s exact test of proportions was used to compare them by milk type (organic *v*. conventional). The levels in each sample were also compared with existing federal tolerance limits^(^^14^^,^^21^^)^. Due to deviations from a normal distribution, median levels of all chemicals of interest were reported for each milk type. Samples with values below the LOD were imputed as one-half the LOD of the laboratory method used for each chemical^(^^43^^)^. Comparisons between median values of organic *v*. conventional milk were done using Wilcoxon rank-sum tests. To facilitate comparison with an earlier study^(^^24^^)^, the analysis for antibiotic and growth hormones was repeated to obtain adjusted least-squares means controlling for percentage of milk fat and region using generalized linear models. All statistical analyses were performed using the statistical software package SAS version 9.4 and a two-sided *P* < 0·05 was considered statistically significant.

## Results

A total of sixty-nine samples were collected (thirty-four organic and thirty-five conventional). Eight samples from seven of the nine study regions were available for analysis. This included three different brands of organic 2 % milk, three different brands of conventional 2 % milk, and one brand each of organic and conventional whole milk. From one region (New York) only two of the three requested samples of 2 % conventional milk were available for analysis and from another (Rocky Mountain) only one of the three requested samples of 2 % organic milk were available (Table 1). A total of ten different brands of organic (with the number of samples per brand ranging from one to eleven) and eighteen different brands of conventional milk (with the number of samples per brand ranging from one to six) were collected (not shown). Of the ten different organic brands tested, four were purchased in multiple regions, including two brands that were purchased in three different regions, one in eight and one in all nine regions. Fewer of the eighteen conventional brands were purchased in more than one region, including one brand purchased in two regions and two brands purchased in three different regions.

### Pesticide results

Residues of several currently used pesticides, including atrazine, chlorpyrifos, cypermethrin, diazinon, hexachlorobenzene and permethrin, were detected in many of the conventional milk samples (26–60 %) but in none of the organic samples (Table 2). Pesticide levels in the conventional samples were below the FDA limit for all with established limits (Table 3). All samples (conventional and organic) were free of detectable levels of the pesticides dicofol, endosulfan-α, chlorthalonil, fonofos, cyfluthrin and fenvalerate. Legacy pesticides, those now prohibited but that remain environmentally persistent^(^^44^^)^, hexachlorobenzene and ppDDT, and the DDT metabolite/degradant, ppDDE, were detected in nearly all of the organic as well as the conventional samples (91–100 %; Table 2). ppDDT was the only pesticide to have a median level that was not statistically significantly higher in conventional compared with organic samples (*P* = 0·38).

**Table 2:** Percentage of samples with detectable levels of pesticide and antibiotic residues by production method (conventional *v*. organic) in the set of retail milk samples collected in nine continental US regions, August 2015

	Conventional (*n* 35)		Organic (*n* 34)		*P* value*
	*n* detected	%	*n* detected	%	
Pesticides*					
Hexachlorobenzene	35	100	34	100	–
ppDDE	35	100	34	100	–
ppDDT	32	91	32	94	1·00
Atrazine	9	26	0		0·002
Diazinon	21	60	0		<0·0001
Chlorpyrifos	20	59	0		<0·0001
Cypermethrin	17	49	0		<0·0001
Permethrin	16	46	0		<0·0001
Dicofol	0		0		–
Endosulfan-α	0		0		–
Chlorthalonil	0		0		–
Fonofos	0		0		–
Cyfluthrin	0		0		–
Fenvalerat	0		0		–
Antibiotics					
Penicillins					
Carbenicillin	0		0		–
Amoxicillin	15	43	0		<0·0001
Tetracyclines					
Oxytetracycline	21	60	0		<0·0001
Sulfonamides					
Sulfamethazine	13	37	0		0·0001
Sulfabromethazine	0		0		–
Sulfadimethoxine	18	51	0		<0·0001
Sulfapyridine	0		0		–
Sulfathiazole	9	26	0		0·002
Ionophores					
Monensin	0		0		–
Lasalocid	0		0		–
Pyrimidine inhibitor					
Trimethoprim	0		0		–

* *P* value for conventional *v*. organic: Fisher’s exact test of proportions (*P* < 0·05 considered statistically significant).

**Table 3:** Medians and ranges for pesticide and antibiotic residues by production method (conventional *v*. organic) in the set of retail milk samples collected in nine continental US regions, August 2015

	EPA limit for milk (pg/ml)	Conventional (*n* 35)		Organic (*n* 34)		*P* value*
		Median (pg/ml)	Range (pg/ml)	Median (pg/ml)	Range (pg/ml)	
Pesticides						
Atrazine†	20 000	<0·01	<0·01–0·29	<0·01		0·0018
Hexachlorobenzene	NA	236	49·0–553	45·5	23·0–225	<0·0001
ppDDE	NA	465	206–694	303	110–492	<0·0001
ppDDT	NA	51·6	<5–137	45	<5–131	0·38
Diazinon†	NA	29·0	<5–150	<5		<0·0001
Chlorpyrifos†	10 000	112	<20–319	<20		<0·0001
Cypermethrin†	100 000	<20	<20–210	<20		<0·0001
Permethrin†	880 000	<5	<5–184	<5		<0·0001
Dicofol	–	<5		<5		–
Endosulfan-α	–	<5		<5		–
Chlorthalonil	–	<5		<5		–
Fonofos	–	<5		<5		–
Cyfluthrin	–	<5		<5		–
Fenvalerate	–	<5		<5		–
	FDA limit (ng/ml)	Median (ng/ml)	Range (ng/ml)	Median (ng/ml)	Range (ng/ml)	
Antibiotics						
Penicillins						
Carbenicillin	–	<1		<1		–
Amoxicillin†	10	**<1**	**<1**–**10**·**2**	<1		<0·0001
Tetracyclines						
Oxytetracycline†	300	14·6	<5–147·2	<5		<0·0001
Sulfonamides						
Sulfamethazine†	0	<1	<1–6·8	<1		<0·0001
Sulfabromethazine	10	<1		<1		–
Sulfadimethoxine†	10	1·2	<1–7·2	<1		<0·0001
Sulfapyridine	0	<1		<1		–
Sulfathioazole†	0	<1	<1–6·0	<1		0·002
Ionophores						
Monensin	–	<10		<10		–
Lasalocid	–	<10		<10		–
Pyrimidine inhibitor						
Trimethoprim	–	<1		<1		–

EPA, US Environmental Protection Agency; NA, not applicable; FDA, US Food and Drug Adminisitration; LOD, limit of detection.

Samples with values <LOD are indicated with ‘<Y’ where Y is equal to the limit of detection for that chemical. Estimated values for samples <LOD were imputed at ½ × LOD for purposes of testing the differences between groups.

Results presented in bold indicate those antibiotics and pesticides for which the residue levels in at least one milk sample exceeded federal limits.

* *P* value for conventional *v*. organic: Wilcoxon rank-sum test (*P* < 0·05 considered statistically significant). Wilcoxon-rank sum tests for differences in population mean ranks (distributions) and is not a median test (note significantly different samples with the same median values).

† Percentage of the total sample below the LOD is >50 %; refer to Table 2 for comparisons of conventional *v*. organic samples using proportions detected instead (recommended by Helsel^(43)^).

### Antibiotic results

The numbers of organic and conventional milk samples with detectable levels of antibiotic residues are presented in Table 2. While residues of at least one antibiotic were found in most of the conventional milk samples (60 %), none were detected in any of the organic samples. The median and range for all antibiotics tested are reported in Table 3. Estimated median levels of amoxicillin, oxytetracycline, sulfamethazine, sulfadimethoxine and sulfathiazole were all statistically significantly higher in conventional compared with organic milk (*P* < 0·0001 to *P* = 0·0018). One of the thirty-five conventional samples (3 %) had an amoxicillin residue level of 10·2 ng/ml, exceeding the FDA limit of 10·0 ng/ml^(21)^. In addition, residues of two sulfonamides with a zero tolerance level for use in lactating cattle, sulfamethazine and sulfathiazole, were detected in 37 % and 26 % of the conventional milk samples, respectively.

### Hormone results

Levels of bGH and IGF-1 by milk production method are presented in Table 4. Median levels in conventional milk samples were 9·8 ng/ml for bGH and 3·5 ng/ml for IGF-1, approximately twenty and three times higher (*P* < 0·0001), respectively, than the 0·5 ng/ml and the 1·1 ng/ml in the organic samples. Results of the sensitivity analysis adjusting for percentage of milk fat and region were similar to those obtained in the primary analysis. The adjusted least-squares mean (95 % CI) for bGH was 9·4 (8·2, 10·7) ng/ml and for IGF-1 it was 3·9 (3·1, 4·6) ng/ml and both still differed significantly from their organic counterparts (*P* < 0·0001).

**Table 4:** Medians and ranges for hormone levels by production method (conventional *v*. organic) in the set of retail milk samples collected in nine continental US regions, August 2015

Hormones	FDA limit	Conventional (*n* 35)		Organic (*n* 34)		*P* value*
		Median (ng/ml)	Range (ng/ml)	Median (ng/ml)	Range (ng/ml)	
bGH	NA	9·8	0·6–17·0	0·5	<0·4–4·6	<0·0001
IGF-1	NA	3·5	0·3–8·1	1·1	<0·1–3·8	<0·0001

FDA, US Food and Drug Adminisitration; bGH, bovine growth hormone; IGF-1; insulin-like growth factor 1; NA, not applicable; LOD, limit of detection.

Estimated values for samples <LOD were imputed at ½ × LOD for purposes of testing the differences between groups.

* *P* value for conventional *v*. organic: Wilcoxon rank-sum test (*P* < 0·05 considered statistically significant).

## Discussion

These results demonstrate that residues of some antibiotics and current-use pesticides were prevalent in the conventionally produced but not the organically produced milk samples collected from retail sites across the USA. Our findings also demonstrated significantly higher levels of growth hormone in the conventional samples. Pesticide and antibiotic levels detected were within existing federal tolerance limits with some important exceptions. One of the eleven antibiotics tested, amoxicillin, had residues exceeding the limit in one (3 %) of the conventional samples and residues of two sulfonimides prohibited for use in lactating cattle, sulfamethazine and sulfathiazole, were detected in a number of these samples (26%–37%).

While none of the organic milk samples had detectable levels of current-use pesticides, most of the conventional samples did. Our results contrast with those reported by the FDA in 2015. Although in that study the findings for milk were combined with those for eggs, only 2·6 % of the thirty-nine ‘dairy and egg’ samples tested had detectables levels of pesticides^(^^45^^)^. It is important to note that the levels of detection for the laboratory methods used were not reported in the FDA study, which compromises our ability to draw comparisons. No recent studies examining pesticide levels relative to limits, or providing a comparison between levels in organic and conventionally produced milk, have been identified.

Although no detectable levels of current-use pesticides were found in any of the organic milk samples, residues of three legacy pesticides were found in nearly all samples, organic and conventional. This included the organochlorines ppDDT, ppDDE (a metabolite and environmental degradate of ppDDT) and hexachlorobenzene, with levels of the latter two significantly higher in conventional compared with organic milk. While the US Department of Agriculture standards for organic feed production prohibit the use of synthetic pesticides on the land for 3 years prior to certification^(^^46^^)^, the half-life of some of these pesticides, such as the once commonly used organochlorines, is 15 years^(^^47^^)^. This persistence in the soil on which food for organic milk-producing cows is grown could be the mechanism through which they are exposed to these pesticides.

In regard to the testing for antibiotics, our results demonstrated that over one-third of the samples exceeded the zero tolerance limit for sulfamethazine and/or sulfathiozole. One sample (3%) exceeded the limit for amoxicillin. This compares to the results of a study done by the FDA in 2015 in which <1 % of the 1918 samples exceeded federal limits for the antibiotics tested^(^^23^^)^. Testing for amoxicillin was not done as part of the FDA study but sulfamethazine residues were detected in 1 of the 953 samples indicating some improper use in lactating cattle. Though oncewidely used to treat bacterial infection in humans, increasing resistance to sulfonamides has led to a decrease in their effectiveness and their medical usefulness in recent years^(^^48^^)^. Amoxicillin is a β-lactam antimicrobial agent in the family of penicillins, which are known allergens for a large proportion (as high as 10 %) of the population^(^^19^^)^. While the results of the previous FDA study^(23)^ demonstrated that, with a few exceptions, antibiotic residue levels were within federal safety limits, our finding that multiple samples exceeded them suggests that further strengthening of the monitoring system is needed to ensure the continued safety of the milk supply. Our analyses comparing antibiotic levels between organic and conventional milk samples demonstrated significantly lower levels in organic milk, which contrasts with the findings of a 2006 study done by Vicini *et al.* In that study, no detectable levels of antibiotics were found in either the organic or the conventional milk samples collected from all US states by employees of the Monsanto Corporation^(^^24^^)^. The levels of detection for the laboratory analysis methods used in that study were not reported.

As growth hormones are produced naturally by dairy cattle, some level of bGH is to be expected in all milk samples, whether produced organically or using conventional methods. We found that the levels of bGH and IGF-1 in conventional milk were significantly greater than those in the samples produced organically. Median bGH levels were 9·8 ng/ml in conventional milk and 0·5 ng/ml in organic milk (*P* < 0·0001). These levels are substantially higher than those reported by Vicini *et al.* in their 2006 study of retail milk. In that study, geometric mean (se) bGH levels, adjusted for milk fat percentage and region, were 0·005 (0·002) ng/ml in conventional milk and 0·002 (0·001) ng/ml in organic milk^(^^24^^)^. When we repeated our analysis to obtain the adjusted geometric mean as was done in the earlier study, the results closely approximated those obtained in our primary analysis. The higher recombinant growth hormone levels in conventional *v*. organic samples calls into question the statement in the 2014 WHO review on drug residues that cited similar concentrations of bovine somatotropin (bGH) in the milk of recombinant bovine somatotropin (rbGH)-treated and untreated cattle as evidence in support of the safety of rbGH^(^^28^^)^. Despite the higher levels of recombinant growth hormone in conventional v. organic milk and in the present compared with the previous retail study, it is imporant to note that the recent WHO review highlighted how, due to species differences in hormone receptors and the impact of degradation during digestion, orally consumed rbGH is unlikely to have a biological impact in humans^(^^28^^)^.

While the magnitude of the difference was lower, we also found IGF-1 levels to be higher in the conventional *v*. organic milk samples, 3·2 times higher in conventional compared with organic milk, 3·5 *v*. 1·1 ng/ml (*P* < 0·0001). Our results are consistent with those reported by Vicini *et al.* showing significantly higher adjusted least-square mean IGF-1 levels in conventional, 3·1 ng/ml, compared with organic milk, 2·7 ng/ml (*P* = 0·001)^(^^24^^)^, as well as with those reported in a 1989^(^^48^^)^ study conducted by Prosser *et al.* in which the concentrations of IGF-1 in the milk treated with recombinantly derived bGH increased 3·6 times after 7 d of treatment, from a mean of 0·44 nmol/l (0·18 ng/ml) to 1·6 nmol/l (0·64 ng/ml)^(^^49^^)^. Despite the observed higher concentrations of IGF-1 in milk produced using conventional *v*. organic methods, a recent WHO review suggests that these higher levels would be expected to have little impact as people naturally produce IGF-1 at much higher levels. Adults produce 10 mg/d^(^^28^^)^ and maintain a mean plasma IGF-1 concentration in the range of 120–460 ng/ml^(^^48^^)^.

### Strengths and limitations

To our knowledge, the present study is the first to compare levels of pesticides in the US milk supply by production method (conventional *v*. organic). It is also the first in a decade to measure antibiotic and hormone levels and compare them by milk production type. Our use of highly sensitive and specific laboratory methods provided valid estimates of the chemicals of interest. In addition, our analysis focused on milk with 2 % milk fat – the type most commonly consumed in the USA – and tested samples collected from retail stores across the country. We were also able to control for seasonal variations in feeding practices by collecting all samples during the same summer month (August) in the same year. Finally, while there were some chemicals where most but not all of the samples had no detectable values (as high as 87 % of the samples), the availability of a single reporting level (or LOD) per chemical allowed us to use suitable non-parametric methods to test differences among groups of interest^(^^43^^)^. These ranking methods will not give a false positive as using equal ranks for values censored as ½ × LOD allowed us to state no more than what is known. For chemicals with <50 % LOD, using the rank-sum test and reporting median/range values (with the <LOD) allowed us to capture the ordering in our data and prevent any loss of information from dichotomizing the data into percentage detected *v*. non-detected.

The study also had important limitations. The laboratory methods used to assess the levels of antibiotics, pesticides and hormones differed from those used in previous studies^(^^22^^)^. As a result, comparisons between studies must be made with caution. In addition, the lack of a laboratory method capable of differentiating the synthetic from the naturally occurring growth hormone limited us to an examination of total bGH levels. The difference between levels in conventional and organic milk was used as an estimate of the synthetic rbGH. Also, the LOD for some chemicals, specifically the ionophores, is as high as 10 ng/ml, compared with 1 ng/ml or lower for others. As a result, it is possible that there are differences in the level of these pesticides between conventional and organic milk that we were unable to detect. While the use of a convenience-based sampling methodology limits our ability to generalize our findings, the fact that milk samples were purchased from local retail stores throughout the country suggests that the results are reflective of milk commonly being consumed. Due to the small number of samples we were unable to examine regional differences that may have provided insight into local factors that influence the use of production-related chemicals. Similarly, we were not able to compare levels by the fat content of the milk samples. Finally, the study design did not allow for an assessment of the health impact of human exposure to the production-related chemicals in milk.

## Conclusion

Residues of current-use pesticides and antibiotics appear to be common in conventional but not organic milk sold through retails stores across the USA, in multiple cases exceeding federal tolerance limits. Similarly, growth hormone and IGF-1 levels were several times higher in conventional milk, which suggests that the difference reflects the use of synthetic growth hormones. While further research is needed to understand the lifetime risk, if any, to milk consumers resulting from their exposure to these chemicals, the present study’s findings suggest that choosing to consume milk produced organically would minimize exposure and any possible associated risks.
